# Supplementary material for: Skilled motor control of an inverted pendulum implies low entropy of states but high entropy of actions
Source: PLoS Comput Biol. 2023 Jan 6;19(1):e1010810. doi: 10.1371/journal.pcbi.1010810 (PMC9851554; doi:10.1371/journal.pcbi.1010810)
Supplement: S1 File — This document contains an additional numerical study based on a computational model of information-theoretic bounded rationality (i.e., the Relevant Information formalism) that supports the routinization hypothesis for the pendulum balancing task presented in this paper. The reported relevant information policy has low action entropy for high levels of skill. This shows that in principle the routinization hypothesis, although confuted by our study on human motor control, has a certain degree of plausibility for the pendulum balancing task, highlighting the biological significance of our findings. (PDF) [file pcbi.1010810.s001.pdf]

Supplementary Material - Skilled motor control of an inverted pendulum implies low entropy of states but high entropy of actions

Nicola Catenacci Volpi[1,*], Martin Greaves[1], Dari Trendafilov[2], Christoph Salge[1], Giovanni Pezzulo[3], and Daniel Polani[1]

[1]Department of Computer Science, University of Hertfordshire, College Lane, Hatfield, AL109AB, United Kingdom
[2]Institute for Pervasive Computing, Johannes Kepler University, Science Park 3, Altenberger Str. 66, Linz, 4040, Austria
[3]Institute of Cognitive Sciences and Technologies, National Research Council, Via S. Martino della Battaglia 44, Rome, 00185, Italy
[*]Corresponding author, n.catenacci-volpi@herts.ac.uk

1 Plausibility of the routinization hypothesis

To show that our findings in terms of large action entropy for competent participants reflect characteristics of human motor control and not just of the structure of the task, in this section we will show that routinization, although confuted by the results of our human experiment, is at least plausible as an hypothesis when considering inverted pendulum control. To this aim, to control the pendulum here we employ a computational model of information-theoretic bounded rationality: the *relevant information* (RI) formalism [1, 2, 3]. The RI artificial controller conforms to the routinization hypothesis: high levels of skill are indexed by low action entropy, with these two quantities being characterised by an inversely proportional relationship.

Given a task, its "relevant information" $I_{\bar{Q}}$ is the minimum information about the state space that an agent needs to know in order to act achieving a given level of performance $\bar{Q}$. The "relevant information controller" is the policy $\pi_{\bar{Q}}$ that attains this minimum amount of information $I_{\bar{Q}}$. The RI policy is in general stochastic, where $\pi_{\bar{Q}}(a|s)$ indicates the probability of selecting action a in state s. A policy that uses the same action distribution in every state has zero RI as the agent does not need to know its state in order to follow this policy. On the contrary, the RI is maximal when the policy has a different action distribution in every state and an agent executing this policy needs to know its state precisely. The RI formalism can be used to study the fundamental trade-off found in a natural organism between efficiency (i.e. minimization of cognitive burden to not violate the organism's limited information processing

capacity) and accuracy (i.e. attainment of a certain performance to achieve a certain goal)[3].

Formally, given the MDP defined in the main text, the RI controller is a policy $\pi_{\bar{Q}}$ that attains the minimum mutual information between state and action $I(\hat{S};A)$ necessary to achieve a given level of average utility $\bar{Q}$. Here, to measure utility we use the MDP action value function $Q^{\pi}(\hat{s},a)$ of a policy π, i.e. $Q^{\pi}(\hat{s},a) \doteq \mathbb{E}_{\pi}[\sum_{k=0}^{F} r(\hat{s}_{t+k+1})|\hat{s}_t = \hat{s}, a_t = a]$, where the expected value is taken over all the trajectories generated using the policy π [4]. Similarly, we can define the state value function $V^{\pi}(\hat{s})$ of a policy π as $V^{\pi}(\hat{s}) \doteq \mathbb{E}_{\pi}[\sum_{k=0}^{F} r(\hat{s}_{t+k+1})|\hat{s}_t = \hat{s}]$. The relevant information can be defined by the following constrained optimization problem

$$\begin{aligned} I_{\bar{Q}} \doteq \min_{\pi(a|\hat{s})} \quad & I(\hat{S};A) \\ \text{s. t.} \quad & \mathbb{E}[Q^{\pi}(\hat{s},a)] = \bar{Q} \end{aligned} \tag{1}$$

where in $\mathbb{E}[Q^{\pi}(\hat{s},a)]$ the expected value is taken with respect to the state and action joint distribution, i.e. $\mathbb{E}[Q^{\pi}(\hat{s},a)] = \sum_{\hat{s},a} P(\hat{s},a)Q^{\pi}(\hat{s},a)$. This optimization is similar to the rate-distortion problem used in information theory for source coding [5]. For this reason, it can be solved with an iterative Blahut Arimoto-like algorithm [3].

To show that the RI controller of the pendulum balancing task conforms to the routinization hypothesis, we computed the RI policy $\pi_{\bar{Q}}$ of the deterministic MDP $(\hat{\mathcal{S}}, \mathcal{A}, T, r)$ reported in the main text with no observation noise for different levels of performance $\bar{Q}$. For this numerical study, we measured the controllers' skill levels by the target action value $\bar{Q}$. Similar results were obtained using the value of the starting state of the experimental trials as a measure of skill level, i.e. $V^{\pi_{\bar{Q}}}(\hat{s}_0)$, with $\hat{s}_0 = (0,0)$ being a still pendulum in a downward position. Furthermore, we take advantage of the explicit probabilistic representation of the stochastic policy $\pi_{\bar{Q}}$ to directly compute the action entropy $H(A|\hat{S})$ and the mutual information $I(\hat{S};A)$ - employing the more accurate equations of the main text (6) and (7) rather than the data-driven estimation used in the experiments reported there.

In Figure 1.a, we report the average utility $\bar{Q}$ as a function of the action entropy $H(A|\hat{S})$ for the pendulum with length $L = 0.6$m. We did not include the results related with other pendulum lengths because these do not provide additional insights. The plot shows that the relevant information controllers fulfil the routinization hypothesis, i.e. large values of utility are indexed by low values of action variability. To illustrate the reported reduction of action entropy in more detail, in Figure 2 we report the probability distributions of actions r and l in $\pi_{\bar{Q}}(a|s)$ for four values of $\bar{Q}$. In Figures 2.a,e the optimization is dominated solely by information minimization and has no incentive in reward maximization ($\bar{Q} = -19.25$). The resulting policy has uniform action distribution and consequently maximum action entropy ($H(A|\hat{S}) = 1.58$ bits). This corresponds to $I_{\bar{Q}} = 0$ bits, represented by the leftmost data point of the mutual information/utility trade-off curve of Figure 1.b. In Figures 2.b,f we can see that due to a slight increase of the target utility ($\bar{Q} = -17.33$) the action variability decreases to $H(A|\hat{S}) = 1$ bit and a regular pattern appears within the action distribution of $\pi_{\bar{Q}}(a|s)$. In Figures 2.c,g, the action distribution is more routinised ($H(A|\hat{S}) = 0.62$ bits) due to the necessary increase of coordination between

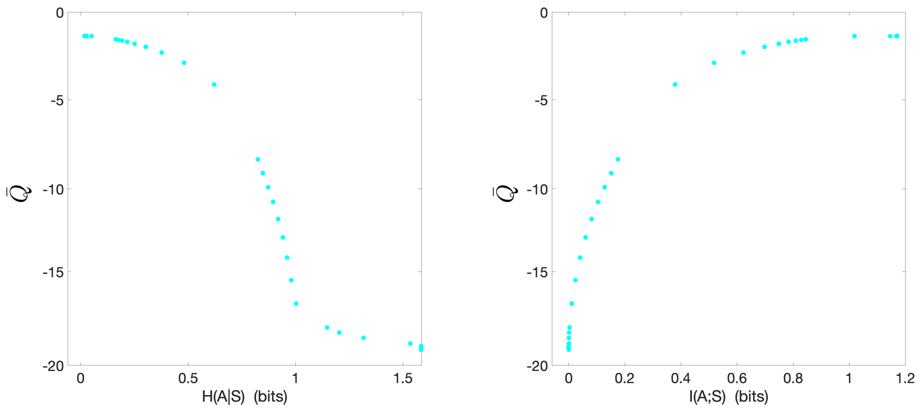

(a) **(b)**

Figure 1: Target action value $\bar{Q}$ as a function of $H(A|S)$ in **(a)** and of $I(A;S)$ in **(b)**, achieved by the corresponding RI controllers.

states and actions required to reach an average target performance of $\bar{Q} = -4$. Although the corresponding increase of RI ($I_{\bar{Q}} = H(A) - H(A|\hat{S}) = 0.38$ bits; see also Figure 1.b), its underlying minimization still results in some uncertainty regarding the RI choice of action in every state. Finally, by imposing a more stringent utility constraint ($\bar{Q} = -1.07$), we encounter a further reduction in action variability, resulting in a $\pi_{\bar{Q}}$ that is close to be fully deterministic ($H(A|\hat{S}) = 0.02$ bits). This policy achieves the maximum achievable average utility and RI, corresponding to the largest data point of Figure 1.b ($I_{\bar{Q}} = 1.17$ bits).

This numerical study showed that although large levels of skill of *human* pendulum control are characterised by large action action variability, in general routinization in this task is possible. Furthermore, the mutual information/utility curves of the RI and human controllers have opposite trends. This shows that human pendulum control is far from being optimal in terms of $I(\hat{S}; A)$ minimization, because, by definition, this implies a monotonic increasing relationship between information and performance. Finally, we found that the RI controllers confirm the inversely proportional trend between $H(S)$ and utility seen so far (not reported because does provide additional insights), implying that they fulfil both the PCT and routinization hypotheses and that in principle there is no reason to assume that these hypotheses are mutually exclusive, as found in the analysis of the human experiment.

References

[1] Daniel Polani, Thomas Martinetz, and Jan Kim. An information-theoretic approach for the quantification of relevance. In *European Conference on Artificial Life*, pages 704–713. Springer, 2001.

[2] Daniel Polani, Chrystopher L Nehaniv, Thomas Martinetz, and Jan T Kim. Relevant information in optimized persistence vs. progeny strategies. In *In:*

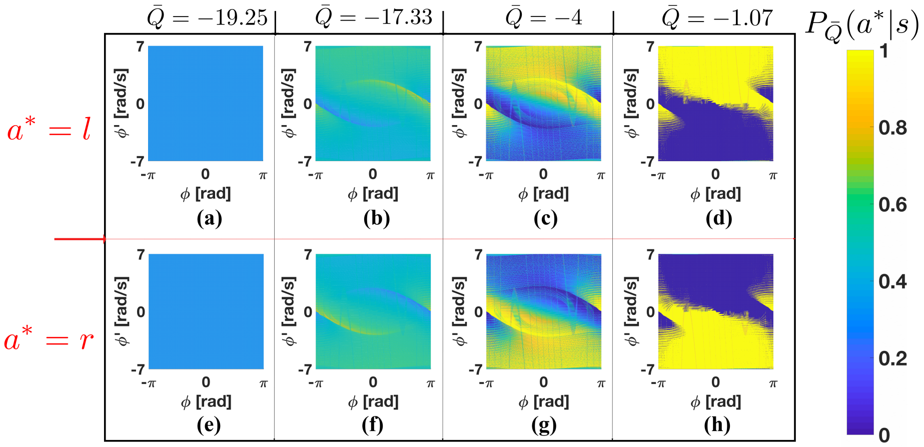

Figure 2: Probability distributions of the RI policies $\pi_{\bar{Q}}(a|s)$ for four target action values $\bar{Q}$.

Artificial Life X: Proceedings of the Tenth International Conference on the Simulation and Synthesis of Living Systems. Mit Press, 2006.

[3] Sander Gerrit Van Dijk. Informational constraints and organisation of behaviour. 2014.

[4] Richard S Sutton and Andrew G Barto. *Reinforcement learning: An introduction*. MIT press, 2018.

[5] Thomas M Cover. *Elements of information theory*. John Wiley & Sons, 1999.
