# Supplementary material for: Skilled motor control of an inverted pendulum implies low entropy of states but high entropy of actions
Source: PLoS Comput Biol. 2023 Jan 6;19(1):e1010810. doi: 10.1371/journal.pcbi.1010810 (PMC9851554; doi:10.1371/journal.pcbi.1010810)
Supplement: Attachment — Submitted filename: rebuttal.docx [file pcbi.1010810.s004.docx]

Dear Editor

We thank you for the opportunity to re-submit a revised version of our manuscript to PLOS Computational Biology. We also thank the reviewers for their commitment and the provided feedback, which we found very useful to improve the quality of the paper. Below, we replied to the points raised by the reviewers and indicated how we modified the manuscript accordingly. In the following, we report in *red and italic* font the comments of the reviewers, whereas our replies are written in regular font. Regarding some very minor changes in the manuscript, such as typos or similar, they are not in highlighted in the revised paper. Also, line numbers refer to the new revised version of the manuscript.

-REVIEWER 1

*“The title suggests that low state entropy and high action entropy are always implied by skilled motor control and hence serve as a general principle. However, this is most likely not always the case, as the authors themselves admit in their discussion when citing previous studies that have investigated different kinds of variability modulation during motor learning including noise reduction [e.g. ref 41].”*

*“Also, the analysis does not clarify the generality of the proposed principle, i.e. in how far the results depend on the particular task chosen.”*

We agree with the reviewer that the relationships between state and action entropies and skilled motor control are not universal, but rather highly task dependent. We also agree that the previous version of the manuscript might have been misleading in this regard. We have modified several parts of the text to remove any misunderstanding regarding the generality of our findings (e.g., lines 29, 43, 47, 107, 108, 112, 113, 412-414, 600, 601, etc.).

In addition to making most of the statements explicitly “pendulum-specific”, we changed the title of the paper to “Skilled motor control of an inverted pendulum implies low entropy of states but high entropy of actions”. Furthermore, we included in the Discussion section several additional paragraphs that highlight the task-dependency of our results (lines 680-686; 694-697; 708-713) and discuss possible characterizations of tasks in this regard (lines 713-725).

*“This is also foreshadowed in the classical work of Nicolai Bernstein who proposed already in the 60s after studying the degrees of freedom problem that motor variability is modulated adaptively depending on the stage of learning. Thus, the story of skilled motor behaviour is most probably more complex than simply minimizing state entropy. This could not only be reflected in the title, but also discussed in more detail, for example by applying entropy concepts to the skittles game studied in 41 or something similar.”*

We agree with the reviewer that in the previous version of the manuscript the description of skilled motor control was limited. In the Discussion section of the new version, we have extended our literature review regarding skilled motor control and learning (lines 686-694), highlighting the contribution of these studies and the relations with our findings. We thank the reviewer for pointing out the classical work of Bernstein; we included a reference to this important work in the paper (line 690). In fact, it is an interesting question to gain a fuller understanding on the interplay on when and where noise or active randomness are introduced into the perception-action loop.

*“It seems that small and large pendulum sizes are somewhat similar to different cursor gains on a computer screen, where large gains induce more variability by magnifying natural motor noise.  Could the results just be a consequence of the fact that smaller pendulum sizes lead to the induction of more motor noise into the system? This and similar questions could be investigated by simulation studies with learning controllers that have different noise properties. The authors write that simulation studies are left for the future , but I can see no reason not to include them in the study (many different learning algorithms exist for the inverted pendulum) as they seem essential to judge the generality of the results (also it is pretty standard to include modeling in PLoS Comp Biol).”*

We thank the reviewer for suggesting the use of a computational model to investigate the effect of different noise sources in determining our findings. We have implemented a Markov decision process (MDP)-based artificial controller that solves the same task that the participants have addressed, which considers the presence of noise in different aspects of the tasks. The model and the results of the simulations are reported in Section 5.3. We believe that the model – thanks to the reviewers’ suggestions - considerably improved the contribution of the paper. We found that two kinds of noise are key to reproduce the trends that we observed in human data: the agent’s observation noise and the agent’s transition model noise. Both of them inject motor noise in the choice of the agents, as suggested by the reviewer. The observation noise allowed to fit the participants’ action entropy, whereas the transition noise allowed to get a similar level of minimum state entropy.

Please note that we systematically investigated various controllers. Specifically, we have considered the spectrum between an optimal policy and a completely random policy, but between these two extremes, we modified the optimal behaviour using an epsilon-greedy policy to model different levels of skill. While this is the simplest strategy to consider, as model it displays already essential features of the phenomenology. It will be a matter of future refinements to consider more sophisticated “deskilling” models.

In this context, we would like to mention that, although we believe that to investigate the effect that the pendulum length may have on these noise levels, and on performance in general, is a very interesting issue, we did not include it in the paper. In this regard, our computational model shows that agents that control longer pendulums achieve lower utility than agents that control shorter pendulums (the contrary of what has been found in the human experiment). As we explain in the paper (lines 505-520), this is because shorter pendulums are faster and so they reach the balancing region before than shorter pendulum (our reward function penalises the time spent out of the balancing area). Also, since we used the same amount of noise for all pendulum lengths, the relative noise affecting longer pendulums is larger. A possible way to address this discrepancy would be assigning different levels of noise according to the length or speed of the pendulums. But, since we do not have a concrete hypothesis regarding how to distribute the noise levels according to pendulum or speed, we have decided to leave this aspect to future work.

We also believe that the relation between pendulum length and participants’ performance is related to limited information processing capacity of humans, which would deserve a separate study and is linked to above issue of how to model “deskilling” in a systematic way.

In other words, the main focus of the paper is to establish the relationship between the level of skill and state and action entropy for a pendulum balancing task, rather than study the precise effects of human bounded rationality in controlling the pendulum. Looking at the results obtained with the reported simulations, we suggest that already the simple proposed model was sufficient to explain our present findings. Of course, this does not preclude the endeavour to develop more refined models of noise (both observation and action noise, whether involuntarily or intentionally produced), but is clearly a task for future studies.

-Minor Comments

*“how many male/female participants? Age range?”*

We have included this information in the paper (lines 129-130).

*“Why is Euler integration used instead of a more precise integration method? The pendulum equation is known to have significant integration errors (the undamped pendulum simulated by Euler increases in energy indefinitely). Can integration errors be neglected?”*

The main reason why we chose the Euler method is for simplicity. We are quite aware that we could have used more accurate methods, such as, say, 4^th^ order RungeKutta or symplectic integrators, or even just implicit Euler, but it was not deemed essential for the study and turned out to be fully sufficient for the sake of the experiment. Notably, because we introduced a dampening factor, and due to the simplicity of the system, in this particular case, we do not expect a major difference in results by using a more accurate difference

One further consideration of relevance here was easy reproducibility; the model can be easily reimplemented without relying on an external library or introducing methods that may not be transparent to a “re-implementer” not familiar with numerical integration methods. In the light of this and since the simplicity did not affect its suitability for the task (in principle, one might have even considered video game-type dynamics with even less claim to realism to study the effects suggested), we believe the use of Euler to be justified.

*“in equation (5) and the other equations is the entropy computed for a single trial (i)? I would have thought the entropy over all state trajectories?“*

Each trial represents a different participant and a different pendulum length, so, to show how the entropies depend on different levels of participants’ skill and on task difficulty (i.e. pendulum length), the entropies were computed over the individual trials (this has been made clearer in lines 268-270). There are not enough data for a statistics over the full trajectory.

*“Similarly, what is the probability P(s^i) and how is it computed? I would have thought it is the probability of a single state trajectory?   But they would be continuous, which means that entropy would not be defined (unless it’s differential)? How would this distribution be estimated from finitely many samples?”*

To compute the state probability, we used binning sampling the time series at a fixed sampling rate. It is worth noting that binning was not expected to create too many problems when compared with differential entropy. Indeed, although the differential entropy of the continuous state and actions variables could have been computed, making our analysis independent from the choice of the binning size, it is well known that the differential entropy is difficult to interpret (see the reference cited in line 252). Furthermore, while the entropy obtained by discretising a probability density through binning depends on the size of the bins, the particular choice of the latter shifts the entropy values by only a fixed additive constant; e.g., if the size of the bins is halved, then the entropy increases approximately by one bit (differences may be due to local changes in density or empirical error). Hence, while the choice of binning may change the entropy’s absolute value, when we consider two entropies computed with the same binning, they are fully comparable, because in computing their difference any binning-dependent terms cancel each other out. The same holds for the estimation of the mutual information between states and actions, which is a difference of entropies. So, although in our analysis a different choice of bins’ size would change the absolute values of the data points in the reported scatter plots, the overall trends of the curves would not change. In other words, since the binning is consistent over all the experiments, all that matters is the relative behaviour of the different entropies, while their absolute values are not essential for our study. Finally, as mentioned in the paper (lines 332-337), we also compared the results of our analysis with the Kraskov-Stögbauer-Grassberger (KSG) method for estimating mutual information, which is designed for fully continuous distributions, to verify the correctness of our analysis in terms of mutual information.

*“Can the trajectories be thought of as Markov processes and be quantified by entropy rates?”*

In principle, it is a good idea to consider entropy rates and we could have done that. However, given the available data set, a problem in this regard is that we do not have an ensemble of trajectories that would allow us to clone the system multiple times and obtain an average across several identical processes. In our analysis we pretend to forget about the transient phase. Hence, we could have only computed the entropy rates assuming that the system is in the stationary phase. Having said that, in the paper we have reported the conditional entropy H(A|S), which is entropy of the next decision given the current state. This essentially can be interpreted as an entropy rate per step, because A is the only source of uncertainty of the system and thus that’s what would have determined the entropy rate. Alternatively, we could have considered a continuous time representation, but we have assumed that participants’ decisions are made at every discrete time step. Also, we could have investigated whether participants’ actions depend on the history of states, but we have assumed a fully Markovian system with no memory effect. That being said, these are all highly interesting questions that deserve a future separate study to be properly investigated.

*“The authors reference a toolbox, but these questions should be clarified in the paper, what options exist to quantify entropy for this task etc.”*

We have inserted in the paper (lines 248-266) a summary of what we have written here in the rebuttal.

*“rather than comparing different lengths of pendula, would it not be good to (also) study entropy values in early and late learning for the same pendulum length? Skilled motor behaviour would correspond to late learning and the entropy values could be compared to unskilled early learning.”*

We agree that analysing skill learning is very interesting, but it is beyond the scope of this study (we made this clearer in lines 103-105, 168-171). In the experiment, we included a training session to allow the participants to familiarize with pendulum control, but we did not analyse data from the training session. We made this choice because the participants had a very different starting points in terms of pendulum balancing skills: some were not able to control the pendulum at all, whereas others were already highly performant at the beginning of the training. So, the training session data are not homogeneous, making an analysis of skill learning more complex. We included a training session mostly to minimize any trajectory artifacts from lack of familiarity with the input controls, and to homogenize the skill level of the participants to some degree - so that they could have a similar level of confidence with the task. In other words, we aimed to minimize any drift introduced by learning into the data.

-REVIEWER 2

*“My main issues regard the relative lack of detail regarding how perceptual control systems actually work, …”*

*“Overall, this is a very welcome advance in research methodology to complement the emerging empirical literature supporting PCT, that needs some greater detail and clarity regarding this theory and how it actually 'works'”*

We thank the reviewer for this suggestion and we agree with the reviewer that in the previous version of the paper the level detail describing PCT systems was insufficient. To overcome this problem, we have included in the Introduction of the paper (lines 66-88) a more detailed description of what is PCT and what the main components of a PCT system are.

*“… and in turn a lack of a clear and simple explanation for how these findings were obtained so consistently and clearly.”*

*“The study provides some very convincing tests of the hypotheses made. However, ultimately the test of a theory is whether it enables the construction of models (or robots) that replicate the naturalistic phenomenon they purport to explain. This is how we appreciate the 'truth' of the theories within physics to enable advances in technology, for example.”*

We concur with the reviewer that the first submitted manuscript included interesting findings, but not enough information was provided to explain the underlying causes of what we observed. To gain further insights on the behaviour we observed, we included a computational model, which, for the sake of simplicity, was based on a Markov decision process (MDP). This model helped us understanding the key role of two different sources of noise (in the observation and transition processes) in explaining our findings, see Section 5.3. While we are aware of the formal differences between MDP and PCT controllers, we believe that the MDP model that we used can be conceptually related to PCT, in the sense that in pole balancing, the “reward” area of MDP and the “perceptual target” area of PCT are related. Therefore, our model provides insights that can easily generalize to PCT; indeed, in the text we interpret our findings in the light of the key assumption of PCT that people are controlling their perceptual states. Furthermore, to establish a more direct connection with PCT models, we point to the 4-level perceptual controller for an inverted pendulum realized by Johnson et al (which is more sophisticated than ours, also given the differences in the task settings).

*“In my opinion, the article requires the reproduction of a closed loop PCT model of how an inverted pendulum might be maintained at a vertical state. There are published examples available that use a three of four level hierarchy, but this could be simplified if made explicit.”*

*“Actions in PCT are the control of perceptual input. Therefore, when a human participant moves their arm hand in a certain direction to keep an inverted pendulum stabilised, this itself involves the control of variables that are hierarchically subordinate to the control of the position of the bob.* *These may not be the focus of the study, but in order to construct a model (or a robot; see Johnson et al., 2020) to balance an inverted pendulum, control of these variables need to be included. The definition of action/behaviour as the control of input in PCT needs to be clear in this manuscript.”*

*“Therefore, whilst this study provides good evidence to support PCT, it does not test the principles of PCT in detail. For further discussion of this issue see Huddy & Mansell (2018).”*

We thank the reviewer to have pointed out the suggested PCT model. This is in line with the idea that our findings fit at a high level with the general PCT framework. Meaning that the participants are minimising the state entropy because they want to control where they want to sense the pendulum, minimising a perceptual discrepancy. However, as mentioned previously, at this stage, to understand what humans do, we decided to compare the results of our analysis with a baseline formalism such as MDP. Nevertheless, we completely agree with the reviewer that it would be very interesting to directly compare a PCT model with our data and analysis. Furthermore, it would be even more interesting to compare the findings observed in the new MDP-based study with the proposed PCT model. However, this would imply a degree of uncertainty as to which specific implementation of a PCT model would be the appropriate one to choose. As a consequence, to be sure that the PCT model would match with what we have found in our data analysis, we would need to properly “unpack” the problem. Amongst other, this would require, for instance, to verify if we could find and control the right parameters and actually fit the parameters with the data, and whether these are matchable with our findings at all. We think this is a highly promising idea by the reviewer very much but, in terms of setup, this means that a whole separate study would be necessary which, we feel, goes far beyond the scope of the paper. This allows us to still point to PCT at a conceptual level (lines 66-88) while inviting the reader to refer to the paper suggested by the reviewer to see how an inverted pendulum can be balanced using an existing concrete PCT model (line 86-88, 646-647). We have also added the reference Huddy & Mansell (2018) to the manuscript (line 69).

*“Following PCT, there would be two reasons for the variability in recorded actions in this study, and the diagram above would be necessary to explain them. The first reason is that actions counteract unmeasured disturbances to the movement of the bob which or often from (NCV: "which often form"?) the side effects of the controllers own movements. These counteracting actions seem to be 'variable' but this is only because the disturbances are not measured; in fact they are fairly deterministic consequences of the error from the vertical reference point caused by a disturbance unfolding over time. This is clearly demonstrated by the close correlation between disturbance and action in tracking tasks (for a review, see Parker, 2020).”*

We thank the reviewer for this useful suggestion. Indeed, this comment was one of the things that convinced us that the relationship between utility and action entropy was caused by “actions counteracting unmeasured disturbances”. But, since in our experiment the pendulum dynamics is fully deterministic, these disturbances that act as “side effect to the controller own movements”, rather than being caused by the pendulum movements, must be caused by some aspects of the participants’ behaviour. So, instead of considering disturbances of the pendulum dynamics, we considered unmeasured disturbances of the perception that participants have about the pendulum movements, which - our numerical study shows - influences the participants control strategy. Hence, the disturbance that allowed us to produce similar action entropy trends to those found in the human experiment is the agent’s observation noise (Section 5.3.2). Verifying whether a similar effect could have been encountered with actual disturbances on the pendulum dynamics is an interesting question for future research.

*“The second reason for variability in action will be the effect of truly random trial-and-error changes to the parameters of the human control system (e.g. the gain, input functions) that develop over time during the learning of the task.”*

We agree that analysing skill learning is very interesting, but it is beyond the scope of this study (we made this clearer in lines 103-105, 168-171). In the experiment, we included a training session to allow the participants to familiarize with pendulum control, but we did not analyse data from the training session. We made this choice because the participants had a very different starting points in terms of pendulum balancing skills: some were not able to control the pendulum at all, whereas others were already highly performant at the beginning of the training. So, the training session data are not homogeneous, making an analysis of skill learning more complex. We included a training session mostly to minimize any trajectory artifacts from lack of familiarity with the input controls, and to homogenize the skill level of the participants to some degree - so that they could have a similar level of confidence with the task. In other words, we aimed to minimize any drift introduced by learning into the data.

-REVIEWER 3

-Major Comments

*“One overarching flaw I find in this paper is in how the background is set up. In the Introduction, the authors talked about wanting to understand mastery of skilled behavior, but then quickly launch into two opposing predictions: perceptual control theory, where the goal is to reduce the variance in the states, versus routinization, which is where the variability of actions is reduced to achieve a highly practiced motor skill. However, I don't find that these are two valid opposing hypotheses, because it seems clear and intuitive that the type of control will depend entirely on the task at hand. For example, in the author summary the others talked about skills like tennis. […] In lines 380-384, the authors even admit that this finding (of action entropy being higher in the task) was quite obviously true from the beginning given the task, rendering the exposition of these two “opposing” hypotheses obsolete.”*

*“I would suggest that the authors rewrite the introduction to be more specific about their opposing hypotheses, because the current setup makes the low action entropy hypothesis seem like a straw man especially when the particular task is being introduced.”*

*“Finally, it might be too far-reaching of a claim to say that your results apply to “skilled action control” in general…”*

We agree with the reviewer that the relationships between state and action entropies and skilled motor control are not universal, but rather highly task dependent. We also agree that the previous version of the manuscript might have been misleading in this regard. We have modified several parts of the text to remove any misunderstanding regarding the generality of our findings (e.g., lines 29, 43, 47, 107, 108, 112, 113, 412-414, 600, 601, etc.).

In addition to making most of the statements explicitly “pendulum-specific”, we changed the title of the paper to “Skilled motor control of an inverted pendulum implies low entropy of states but high entropy of actions”. Furthermore, we included in the Discussion section several additional paragraphs that highlight the task-dependency of our results (lines 680-686; 694-697; 708-713) and discuss possible characterizations of tasks in this regard (lines 713-725).

*“tennis is really different than the task in their actual experiment---to perfect a serve in tennis, one has to practice and repeat the serve over and over again to achieve low-variance, stereotyped movements. Yet, in the pendulum balancing task, the goal is to maintain a stable state, and the way to do that is to constantly vary your actions”*

*“Because “state” can often be defined in various ways, one could argue that in the tennis example, the steady-state that is being maintained is the successful serve of the ball. The tennis player must find an action that ensures that the ball will make it over the net every time. The main difference in maintaining this steady state versus the steady state of a pendulum is that while there could be many different actions (e.g., serves) that are successful, it is often most advantageous to perfect one type of serve that works every time, instead of trying out a different serve for each game that is played. But an additional question is whether the final goal state distribution has a high or low entropy: does it matter where exactly the ball lands on the other side, or is a serve successful as long as the ball goes over the net?”*

*“This might be a more interesting hypothesis if there were several tasks that were being studied which varied the entropy of the final goal state.”*

We thank the reviewer for this interesting insight regarding a task characterization that could determine the trends of our information curves in terms of goal state entropy. In Section 6 we discuss about this proposed tasks’ characterizations related to goal state entropy with respect of our findings (lines 713-725). In doing so, we have used the reviewer’s example of tennis (lines 708-713), which we found quite useful.

We believe that an important characterisation in this regard is whether the task is cooperative or antagonistic (with respect to the environment or other agents). In fact, in cooperative tasks often the objective is to reach a set of goal states; whereas in antagonistic tasks usually creating uncertainty is a *desirable* strategy, leading to an as wide as possible target state distribution. Hence, in general, cooperative tasks can be expected to have a smaller goal state entropy than antagonistic tasks. Consequently, also the state and action entropies would differ, making this kind of characterisation an interesting object of study to understand other phenomena that could determine a certain trend of the entropies’ curves. We thus need to treat cooperative and antagonistic scenarios quite differently. In our case, we have only one agent which – naturally – “cooperates with itself”.

The objective of our research is to establish whether humans pay more attention to states or to actions when they must reach a goal state. In our pendulum balancing task, there are certain sequences of states that are required to be traversed to reach the goal, which contribute to the reduction of the state entropy.

However, we agree with the reviewer that, of course, there are other tasks where to traverse certain sequences is not required or, more generally, where there are less constraints to fulfil in order to complete the task. In this case, agents are freer to “spread” the entropy within the state space. To understand more in depth how this may happen represents a different research question from the one addressed in our paper. It would be interesting to find out in a future study if, in a non-antagonistic setup, but with larger tolerance concerning the acceptable states, there would be a preference for reduced state or action entropy or some combination thereof.

*“In section 5.3, I suggest reframing the motivation for doing an analysis of time spent in the balancing region. What question are you trying to answer here?”*

We thank the reviewer to have pointed out that the motivation of this section was missing in the previous version of the manuscript. We have added such motivation in the new version of the paper (lines 371-384).

-Minor comments/Questions

*“223: why did you bin state space into different sizes? 1000 bins for angle interval vs 200 bins for angular speed interval?”*

While the entropy obtained by discretising a probability density through binning depends on the size of the bins, the choice of the latter shifts the entropy values essentially by only a fixed additive constant (e.g., if the size of the bin is halved, then the entropy goes up by approx. one bit). Hence, while the choice of binning may change the entropy’s absolute value, if we consider two entropies computed with the precisely same binning, they are fully comparable, because in computing their difference any binning-dependent terms cancel each other out. The same holds for the estimation of the mutual information between states and actions, which is a difference of entropies. So, although in our analysis a different choice of bins’ size would change the absolute values of the data points in the reported scatter plots, the overall trends of the curves would not change. In other words, since the binning is consistent over all the experiments, all that matters is the relative behaviour of the different entropies, while their absolute values are not essential for our study (furthermore, the chosen binning size gives interpretable results). We note that there is no reason to assume that angle and angular speed should have the same number of bins, because these are different and incomparable dimensions, so their binning can be chosen independently from each other.

*“251: why 270ms after?”*

Thank you for noting this. We have added the following in the paper: “This interval approximates the estimated time that elapses to execute a motor control” (please, see the [reference] cited in lines 300-302).

*“I would recommend citing some more recently work in the motor skill learning literature. See more work by Bence Olveczky’s lab, such as Dhawale et al. 2019, Current Biology. In their study, animals vary their actions to maintain the steady-state of reward, and change their actions when they notice that the reward region has changed.”*

We thank the reviewer to have indicated the work of Dhawale et al. We have included this reference in the manuscript and followed the reviewer recommendation regarding including additional recent work about motor skill learning (lines 685-687).
