# Supplementary material for: Skilled motor control of an inverted pendulum implies low entropy of states but high entropy of actions
Source: PLoS Comput Biol. 2023 Jan 6;19(1):e1010810. doi: 10.1371/journal.pcbi.1010810 (PMC9851554; doi:10.1371/journal.pcbi.1010810)
Supplement: Attachment — Submitted filename: rebuttal.docx [file pcbi.1010810.s005.docx]

Dear Editor

We thank you for the opportunity to re-submit a revised version of our manuscript to PLOS Computational Biology. We also thank the reviewers for their commitment and the provided feedback, which we found very useful to improve the quality of the paper. Below, we replied to the points raised by the reviewers and indicated how we modified the manuscript accordingly. In the following, we report in *red and italic* font the comments of the reviewers, whereas our replies are written in regular font. Regarding some very minor changes in the manuscript, such as typos or similar, they are not in highlighted in the revised paper. Also, line numbers refer to the new revised version of the manuscript.

-REVIEWER 1

*“the authors motivate the study with wanting to distinguish whether motor skilll is rather reflected by routinization (signature: small action entropy) or perceptual control (signature: small state entropy). it seems intuitive that any kind of feedback process (like tracking, pendulum control, etc.) should probably follow the latter. however, the question is what is small and what is large in terms of entropy? the natural answer, it seems to me, would be to study the learning process: which entropy is reduced over time? but instead the authors study different lengths of pendula. so the question is, how does one pendulum length compare with another in terms of the two hypotheses…”*

We agree with the reviewer that to study the learning process of the pendulum balancing task would be very interesting and revelatory with respect to the routinization and PCT hypotheses. Having said that, the focus of our paper has always been pendulum control *after* the learning process has converged. Hence, we do not model progress, but “snapshots” through different skill levels which gives us a well-defined picture of equilibrated skills. In some aspects this is even cleaner than learning models, which have to contend with possibly multiple interacting time scales which are hard to separate. As discussed in the rebuttal of the previous submission, for this reason, the experiment has been designed with a separate training session preceding the actual trials. To study the learning process would require a different design (and to run a separate experiment), which is beyond the remit of the present study. In other words, we take in the reviewer comment on this, but we leave it for future work because it would deserve a distinct study.

We concur with the reviewer that, in absolute terms, it is not entirely obvious to interpret what large and small entropy mean. Therefore, in the paper, to determine which hypothesis hold between routinization and PCT, we investigate how the entropies values change *relative* to the controllers’ skill levels. As alternative to learning progress, in the paper we use two proxies to measure skill level. The primary and more direct way to measure skill is the employed utility function. Then, we considered tasks with different pendulum lengths, which imply different levels of difficulty of the control problem. To make the same participant balance pendulums of different lengths allowed us to explore more levels of skill in the experiments. Thus, by making the pendulum length shorter or longer we probe the space of skills. To show this, and to answer the question “*how does one pendulum length compare with another in terms of the two hypotheses”*, in the new manuscript we added two new figures, one related with the action entropy H(A|S) (Figure 2.b) and one related to the state entropy (Figure 4.b). In these figures we reported the data points of different pendulum lengths in different subplots, reporting the trends of the entropies for each pendulum size separately. We can see the same entropies’ trends for all panels (i.e., higher levels of skill are indexed by smaller state entropy and larger action entropy). These trends have also been quantified in the new Table 1, which reports the Spearman correlations of the utility with H(S) and H(A|S) for every value of L separately, confirming the satisfaction of PCT and the confutation of routinization for each pendulum length. In the two new Figures 3 and 6, we reported the average action and state entropies for each pendulum lengths. Figures 3 shows that longer pendulums imply large average action entropy and small average state entropy, whereas Figure 6 shows that shorter pendulums imply small average action entropy and large average state entropy. We believe this analysis shows the validity of using pendulum length as proxy for skill level and its ability to distinguish between the two hypotheses.

A salient phenomenon that arose from our analysis with respect to pendulum length is the fact that more capable subjects balancing short pendulums, which are more difficult to control, are quite comparable to less skilful subjects balancing longer pendulums, which are easier to control (i.e., they match up and lie on the same utility-entropy surface). We believe this to be quite relevant, because in principle there is a priori no reason to assume that such scaling holds in general. This is something that we suspect may be due to the nature of the pendulum task. But at this stage the precise nature of this phenomenon is under investigation and thus we decided not to discuss it to the full extent it in the paper.

*“…and what do the results shown in Figure 3 actually mean? does it simply mean: controlling a smaller pendulum is more difficult, therefore it is associated with lower utility and higher state entropy. but is this just a consequence of feeding the same amount of noise into different pendula with different lengths?”*

The reviewer’s interpretation of (the old) Figure 3 (now Figure 4 in the new manuscript) is correct. Let’s take the example of longer pendulums. We would expect to see subjects to be more competent when controlling the longer pendulums. This is confirmed by the larger number of data points with large utility in the subplots related with longer pendulums. To satisfy the PCT hypothesis, we would also expect to see more participants controlling the longer pendulums to have small state entropy. This is also confirmed by the subplots of Figure 4. Finally, looking at Figure 6, we can see that longer pendulums have smaller average state entropy. Similar arguments can be made considering shorter pendulums and large state entropy values or considering the action entropy trends.

Regarding the same noise being fed into pendulum of different lengths, it is important to note that there is no noise fed in the pendulum dynamics. The pendulum dynamics employed by the simulator during the experiment is deterministic. Hence, all the uncertainty found by our analysis necessarily comes from the participants’ behaviour. The effects of having controllers with observation noise, and a noisy transition model on the state and action entropies has been investigated in Section 5.3.

*“how would that distinguish between the two different hypotheses from the beginning? this would suggest that the entropy plots are simply a property of the task and do not tell us much about the human controller. is there even a controller conceivable in this task that would formalize the routinization hypothesis? in my mind the cleanest way would be to formalize systematically controllers corresponding to the two hypotheses and study their behavior with pendula of different lengths and confirm that they make different predictions, and that human data fits one of them and not the other (a model-based quantitative comparison).”*

We agree with the reviewer that to show that our study tells us something about human behaviour and does not simply demonstrate certain properties of the pendulum balancing task, is a central issue. On the one hand, as stated multiple times in the paper, we believe that the inversely proportional relationship between state entropy and utility may be independent from the employed control strategy and rather be a consequence of the relationship between the adopted reward function and the pendulum dynamics. On the other hand, since the employed pendulum dynamics is fully deterministic and all the system's uncertainty must come from the participants' decision-making, the study of action variability is informative about certain aspects of human motor control.

In this regard, we added the following text to the manuscript (line 670):

“Figure 11.a shows that in principle multiple solutions of the pendulum balancing task are possible for large utility values (similarly, this is shown in Figures 12.a and 13.a). Some of these solutions have large action entropy, others (in the upper left portion of the plot) have small action entropy. On the contrary, our results show that the solutions adopted by the biological controllers do not actually span this large set of strategies, but only a subset of the possible solutions (i.e., those with large action entropy for large utility). This phenomenon could be explained by the fact that the human controllers operate under sensory uncertainty over the state of the pendulum and its dynamics, as shown by the artificial controllers in Sections 5.3.3 and 5.3.4 respectively. In favour of this interpretation, Figure 13 contains the plots that better reproduce the trend of the human action and state entropies (compare Figures 13.c with Figure 2.a and Figure 13.d with Figure 4.a, respectively). “

Furthermore, we took on board the reviewer’s suggestion to implement an artificial controller that supports the routinization hypothesis. We chose to report this new study in the Supplementary Material, in order to limit the length of the paper and to not complicate further its presentation. This Relevant Information (RI)-based controller shows an inversely proportional relationship between skill level and action entropy (see Figure 1.a of the Supplementary Material). We believe that the of RI policies depicted in Figure 2 of the Supplementary Material show a convincing picture of how routinization may look in terms of action distributions (i.e., how the distribution becomes more structured as the policy becomes more routinised). This text has been added to the main manuscript to link these findings to the Supplementary Material (line 681):

“An alternative interpretation of our results is that the action entropy plots are simply a necessary consequence of the structure of the pendulum task, and it would be impossible to design a controller that formalizes the routinization hypothesis. However, we performed a control simulation (see the Supplementary Material) showing that a controller that formalizes the routinization hypothesis is possible and hence our results indeed reflect characteristics of the human controller and not just of the structure of the task.”

*“minor comment:*

*figure 9 and 10 need the correct lengths in the legend.”*

We thank the reviewer to have pointed out this. We have corrected the legends of the colour bars accordingly.
